# A Topological Sensitive Node Importance Evaluation Method in Aerospace Information Networks

**DOI:** 10.3390/s23010266

**Published:** 2022-12-27

**Authors:** Peng Yang, Shuang Hu, Shijie Zhou, Jiaying Zhang

**Affiliations:** 1 School of Information and Software Engineering, University of Electronic Science and Technology of China, Chengdu 610056, China; 2 School of Mathematical, University of Electronic Science and Technology of China, Chengdu 610056, China

**Keywords:** transfer matrix, spatial information network, invulnerability, node importance

## Abstract

With the rapid development of communication technology and the diversification and rapid growth of network application demands, the requirements for the anti-destruction capability of information networks have also increased. Therefore, an efficient and accurate metric of the survivability of satellite networks has become a hotspot of current research. Firstly, in this paper, we propose a transfer matrix-based spatial information network invulnerability evaluation algorithm. The algorithm draws the idea of a node deletion method to determine the initial importance of nodes and then establishes a formula for the importance transfer probability of the nodes. In addition, an evaluation algorithm of spatial information network invulnerability is obtained using the network structure entropy theory. Simulations show that our new evaluation algorithm based on the transfer matrix is more accurate compared to degree centricity, natural connectivity and other methods in evaluating the importance of the nodes. The proposed index can effectively reflect the change in the network topology and evaluate the network survivability.

## 1. Introduction

In today’s globalized communication era, the ground network cannot meet the communication demands due to natural conditions, therefore the development of a satellite network with global coverage has become an inevitable choice. Compared with terrestrial networks, satellite networks have the advantages of wide coverage and flexible networking [1]. Due to the inherent fragility of the satellite network, nodes or links may fail suddenly. Once a problem occurs, the performance of the entire satellite network may drop sharply or even trigger a domino effect and cause the entire network to be disrupted [2,3,4,5,6]. Considering the high dynamics of node movement in satellite networks and the diverse connections between nodes, the topology of the network is complex and changeable; therefore, it is of great theoretical and practical significance to study the vulnerability of satellite networks.

At present, research on the vulnerability of network topology structures has emerged in endless streams. This research has great significance for improving the robustness of the actual system and for designing an efficient system architecture by evaluating and protecting the important nodes. For example, the jumping-surface node method [7,8,9], the evaluation model based on the mean square error of the node invulnerability metric value [10], the evaluation method based on node deletion [11,12,13,14], the evaluation method based on natural connectivity [15,16], and the mutual information method [17], etc. These models and methods have their own characteristics but these research methods are mainly suitable for static networks and have limitations for satellite networks with high dynamics; the evaluation results are not ideal.

Usually, important nodes play a key role in network functionality and have an important impact on network dynamic processes such as network synchronization and cascading faults [18,19,20,21,22]. Therefore, evaluating the importance of nodes in the network and adopting protection strategies can improve the overall resilience of the network. The importance of a network node is not only related to its local significance but also closely related to its position in the network and the degree of interdependence with other nodes. Based on the above considerations and the node importance contribution, this paper proposes a new method for network node importance evaluation. This method not only considers the contribution of the importance of adjacent nodes and non-adjacent nodes to the evaluated node but also considers the global importance of the node. By combining the global and the local, the node importance evaluation result is more accurate. A metric that characterizes the resilience of the overall network is obtained by using network structure entropy [23,24].

The structure of this paper is as follows. In Section 2, we will introduce related works on general network resilience. In Section 3, we propose the node importance transfer probability matrix model. Section 4 will give the survivability evaluation algorithm and Section 5 will carry out a simulation analysis that justifies the feasibility and effectiveness of our method. Section 6 summarizes the paper.

## 2. Related Work

A spatial information network can be described as a graph G(V,E). The satellite network system is composed of N satellite nodes and inter-satellite links. V={1,2,…,N} represents the collection of satellite nodes and $E=\left\{e_{ij}|i,j\in V,i\neq j\right\}$ represents the collection of inter-satellite links.

If the communication link of two satellites is not blocked by the earth at a certain time and the communication link distance is less than the maximum threshold between satellites, the two satellites are said to be “visible” or “connectable”. Let the adjacency matrix of G(V,E) be $A\in {\mathbb{R}}^{N*N}$, when two satellites i and j are visible, if $a_{ij}=a_{ji}=1$, otherwise $a_{ij}=a_{ji}=0$.

Let $D\in {\mathbb{R}}^{N*N}$ be the shortest path matrix, where $d_{ij}$ represents the shortest path length between satellite nodes i and j.

**Definition** **1.** *Network efficiency [25]. Network efficiency is a metric of the efficiency of information exchange between nodes in a network. In complex networks, network efficiency applies to both local and global scales. On a local scale, it quantifies the network’s ability to resist failures in a small range. Globally, it quantifies the efficiency of the simultaneous exchange of information in the entire network. Its equation is:*

(1) $$E=\frac{1}{N\left(N-1\right)}\underset{i\neq j}{\sum }\frac{1}{d_{ij}}$$

**Definition** **2.** *Degree centrality [26]. Degree centrality characterizes node centrality in network analysis. The greater the node degree means the higher centrality of the node and the more important the node is in the network. The calculation of node degree centrality is as follows:*

(2) $$DC\left(i\right)=\frac{k_{i}}{N-1}$$

*where*$k_{i}$*represents the number of edges connected to node*i.

## 3. Node Importance Evaluation Model

### 3.1. Node Initial Importance

For a connected graph, each node in the graph has a different influence. For example, the root node is often of greater importance than the leaf nodes. Once an important node fails, it impacts the connectivity and robustness of the network. Therefore, we draw the idea of a node deletion method that removes individual nodes in the network in turn and evaluates the changes in network structure and efficiency before and after deletion. Once a node in the network fails, its adjacent edges will also break at the same time and information transmission cannot be carried out at this time. The effect of deleting a node may be very different. If a node is very important, removing it will lead to changes in the network topology and a sharp decline in network efficiency. If it is a terminal node, its removal has almost no effect. Finally, this paper proposes that the initial value $C_{i}$ of the node importance in the network is defined by the following equation:

(3) $$C_{i}=\frac{E\left(G\right)-E\left(G\prime \right)}{E\left(G\right)}$$

Among them, E(G) is the network efficiency and E(G′) the network efficiency after deleting the node. This metric reflects the change in network efficiency after the node is deleted. The larger the change the more important the node is and the importance is quantified from a global perspective.

### 3.2. Node Importance Transfer Probability Matrix

If any node in the spatial information network is isolated, it will inevitably be affected and restricted by adjacent nodes. The interaction between nodes can be described by the probability transfer matrix based on node importance [27].

There are complex dependencies between nodes and the dependence intensity varies with the location of the nodes. For node i, we define its adjacent nodes as the first layer nodes and the nodes with a distance of 2 as the second layer nodes, and so on. Since the importance of node i in the nodes of the same layer is different and the node degree centrality can be used to describe the direct influence of a node in the network, we use the degree centrality to describe the dependence between nodes. The following experiment will show the influence of a node on other nodes.

**Figure 1:**
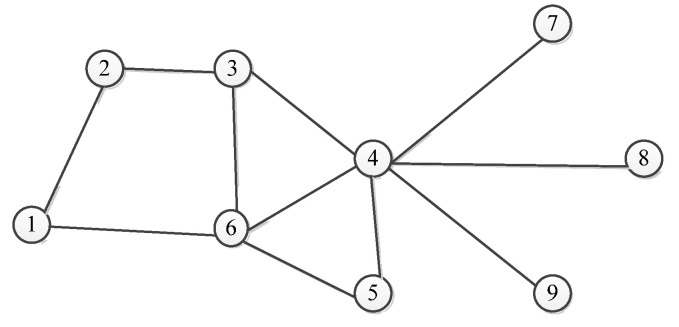
Network topology (Numbers 1–9 represent node numbers in complex networks).

Figure 1 is a typical complex network topology diagram with nine nodes. Node 4 is directly connected to nodes 3–7 and they are all located on the first layer of node 4. We use natural connectivity to calculate the importance of each node. Table 1 shows the changes in the importance of nodes 3–7 before and after deleting node 4.

We can see that although nodes 3–7 are directly connected to node 4, when node 4 fails, the difference in the impact on its adjacent nodes varies, indicating that the contribution of nodes to nodes at different positions on the same layer is different. Therefore, if there is a strong dependency between a node and another node, the importance of the node can be transferred to another node.

The impact of a node failure is not limited to its adjacent nods. Take node 2 in Figure 1 as an example, we can see that node 1 is on the first layer of node 2, and node 4 is on the second layer of node 2. We delete node 1 and node 4 and observe how it affects node 2. The experimental results are shown in Table 2. 

It can be seen from Table 2 that when node 1 fails, the impact value on node 2 is 0.065, and when node 4 fails, the impact on node 2 is 0.095. It can be seen that the influence of node 4 on node 2 is higher than that of node 1 on node 2. The experiment shows that when the nodes have strong reachability, it does not only affect the adjacent nodes but also affects the non-adjacent nodes. For a node, if the influence of non-adjacent nodes is greater than that of adjacent nodes, the importance of non-adjacent nodes cannot be ignored. However, if multi-step non-adjacent nodes are considered, the complexity of the algorithm will increase. Achieving a balance between running speed and accuracy is the key to the problem. Reference [28] found that when considering non-adjacent nodes of order two or above, the accuracy improvement is marginal but the efficiency of the algorithm drops sharply. Therefore, considering the adjacent nodes in the second order can balance the accuracy and time complexity and this paper only considers the second-order neighbors when evaluating the importance of nodes.

Next, we will characterize the importance of dependence between the nodes in the network. In Figure 1, it is not difficult to identify when node 4 is deleted, nodes 7–9 will become isolated nodes, and their importance is 0. However, nodes 3, 5 and 6 are adjacent to other nodes, and their importance will be different and will not be 0. Therefore, if the degree of a node is smaller, it is more easily affected by other nodes. On the contrary, if the node degree is larger, when one of the nodes fails, due to the existence of other adjacent nodes, its importance will not change too much.

Because node i has different important contributions to nodes on the same layer, degree centrality can directly reflect the influence of nodes in the network. In addition, the dependence between nodes is inversely proportional to the shortest distance between nodes. Therefore, we propose the following probability calculation formula of node importance.

**Definition** **3.** *Node importance transfer probability. It reflects the contribution probability of node *i *to node *j *when node *i *assigns its initial importance to all nodes in the second-order domain. Its calculation formula is:*

(4) $$p\left(i,j\right)=\frac{DD\left(i,j\right)/DC\left(j\right)}{\sum_{j\in \tau }\left(DD\left(i,j\right)/DC\left(j\right)\right)}$$

(5) $$DD\left(i,j\right)=\{\begin{array}{c} \frac{1}{d_{ij}}\;\;\;,j\in \tau \\ 0\;\;\;,other \end{array}$$

*where *τ* is the set of adjacent nodes within the second layer and *$d_{ij}$* is the shortest distance between two nodes.*

Since the efficiency reflects the average difficulty from one node to other nodes in the network and is a measurement of the importance of nodes in the global scope, we initialize the importance of node i with the efficiency $C_{i}$ of node i, and then obtain the node importance transfer correlation matrix:

(6) $$H=\left[\begin{array}{cccc} 0 & p_{12}\cdot C_{1} & \cdots & p_{1n}\cdot C_{1}\\ p_{21}\cdot C_{2} & 0 & \cdots & p_{2n}\cdot C_{2}\\ \vdots & \vdots & & \vdots \\ p_{n1}\cdot C_{n} & p_{n2}\cdot C_{n} & \cdots & 0 \end{array}\right]$$

In Equation (6), $H_{ij}$ represents the importance contribution of node i to node j. Considering the global network efficiency and local transfer probability of nodes, the importance $I_{j}$ of node j can be defined as:

(7) $$I_{j}=C_{j}\times \left(\sum_{i=1,i\neq j}^{n}P_{ij}\cdot C_{i}\right)$$

It can be seen that the importance of a node is related to the efficiency of the node itself, its degree centrality, and the distance between the nodes. It integrates the global and local information of the node, which can improve the evaluation accuracy. When the topology structure changes, the distance between the nodes will change accordingly and the evaluation method can effectively and accurately estimate the value of node importance, which meets the needs of the satellite network node importance evaluation.

## 4. Network Survivability Theory

### 4.1. Network Structure Entropy

The satellite network can be built into a graph network model and there are often complex connection relationships between nodes. Essentially, the satellite network belongs to a special scale-free network, which has very few core nodes and a large number of end nodes in the network. Scale-free networks are extremely vulnerable to deliberate attacks. Tan Yuejin and Wu Jun believe that the scale-free characteristic of the network is the “heterogeneity” of the network and that the concept of “entropy” can be used to study this “heterogeneity” of the network [23,24]. Therefore, the two scholars proposed the concept of “network structure entropy”. They believe that the greater the entropy of the network structure, the more uniform the distribution of network nodes, and the weaker the heterogeneity of the network, that is, the fewer core nodes in the network, the stronger the network survivability when the network is attacked, and vice versa.

### 4.2. Satellite Network Survivability Assessment

In Section 4.2, we calculate the importance value $I_{i}$ of each node from the importance transfer matrix, and then the network structure entropy based on the node importance can be expressed as [23,24]:

(8) $$S=-\sum_{i=1}^{N}I_{i}\cdot \ln I_{i}$$

When the importance of nodes in the network is clarified, the higher the importance of the nodes in the network, the more critical the status. For the satellite network topology, when the more critical nodes are attacked, the more destructive it is to the network topology, and the easier it is to cause network interruption. It can be seen that the importance of nodes is closely related to the survivability of the satellite network. If there are no obvious key nodes in the network, the network is more uniform, and the stronger the survivability of the network will be. On the contrary, if there are several obvious key nodes in a network, the network will be more uneven. When the key node is attacked, it has a great impact on the robustness of the network, resulting in poor survivability of the network. Therefore, we can use entropy to evaluate the survivability of spatial information networks.

**Figure 2:**
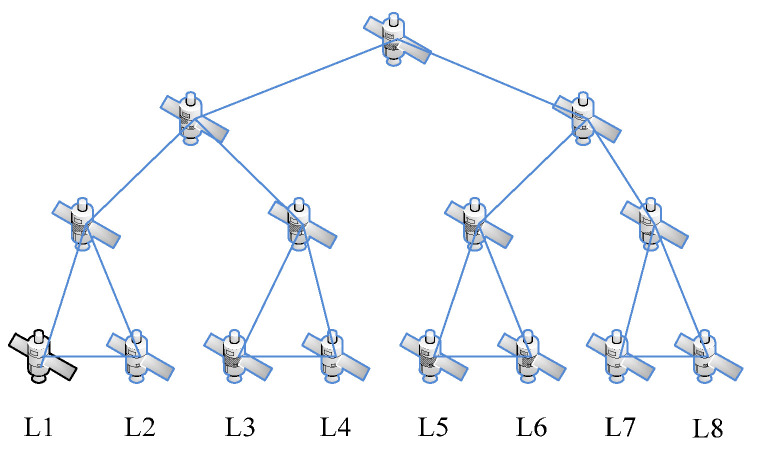
Satellite network (L1–L8 represents the lowest altitude satellite node) (time T1).

**Figure 3:**
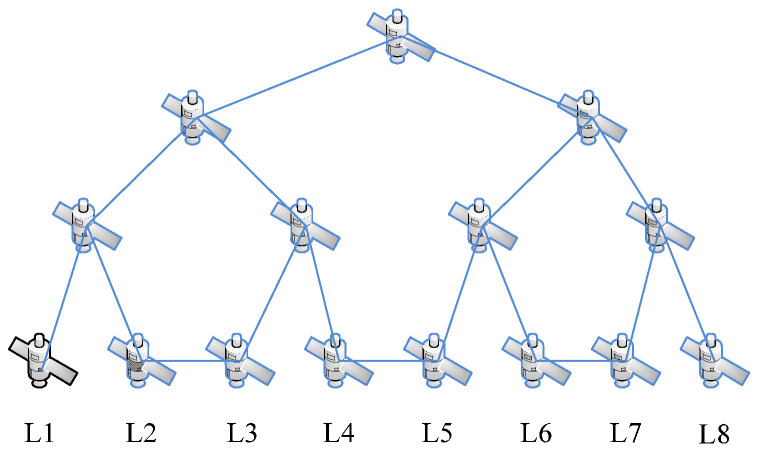
Satellite network (L1–L8 represents the lowest altitude satellite node) (time T2).

In the space information network, nodes represent different types of spacecraft. Due to the different functions and orbits of the spacecraft, the flight speeds of the spacecraft vary greatly. Therefore, the network topology is highly dynamic, that is, the links between the nodes in the network will frequently switch, which will lead to continuous changes in the network topology. In this process, the number of nodes does not change. In view of the appeal, we will carry out simulation experiments based on the fat-tree network. The fat-tree network topology is shown in Figure 2 and Figure 3.

In the process of operation of the spatial information network, the pathway between L1 and L2, L3 and L4, L5 and L6, L7and L8 disappears, and a new pathway is established between L2 and L3, L4 and L5, L6 and L7. This is shown in Figure 3.

## 5. Experimental Simulation and Analysis

### 5.1. Simulation Experiment and Analysis of Node Importance

**Figure 4:**
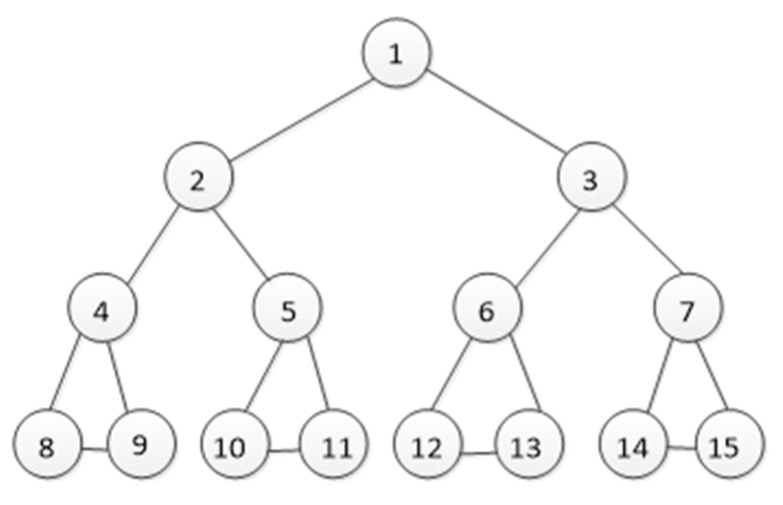
Network topology (Numbers 1–15 represent satellite nodes at different altitudes) (time T1).

**Figure 5:**
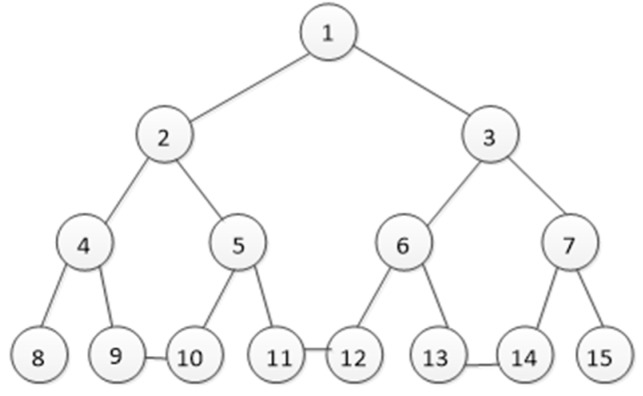
Network topology (Numbers 1–15 represent satellite nodes at different altitudes) (time T2).

We assume that any two satellites within the visible range can have a connecting link. In the case of topological structure and resource constraints, due to the high dynamics of satellite networks, the links between satellite nodes are being disconnected and reorganized frequently, thereby reconstructing the network topology and in the generated topological structure, many topological structures are extremely similar. Therefore, how to effectively distinguish networks with similar topological structures has become the key to evaluating the quality of an index. In this article, we selected two relatively similar topological structures for the experiments and their topological structures are shown in Figure 4 and Figure 5.

#### 5.1.1. Node Importance Algorithm Accuracy Evaluation

On this basis, by comparing the four methods of degree centrality, natural connectivity, jumping-surface node method and mutual information method, the feasibility and effectiveness of the method in this paper are illustrated. Let us take Figure 4 as an example and compare the results of the five sorting methods in Table 3. Since the network is symmetrical, this article only lists some representative node evaluation results, as shown in the following Table.

It can be seen from Table 3 that all methods can evaluate the importance of nodes. Degree centrality is the simplest measurement method, reflecting the importance of nodes in a local range. However, this method cannot distinguish the importance order of nodes, because some nodes have the same degree centrality value, for example, both nodes 2 and 4 are 0.2. We cannot compare other methods intuitively. Therefore, we delete some important nodes and the changes in network connectivity further clarify the performance of natural connectivity, the jump-surface node method, mutual information method and our method in this paper.

Removing nodes from the network will reduce the connectivity of the network and the worst connectivity corresponds to the best sorting method. Connectivity is reflected in two measures: the number of subgraphs and the size of the largest subgraph after decomposition. The larger the number of subgraphs or the smaller the size of subgraphs, the worse the connectivity, which means that the corresponding sorting method has better performance.

**Figure 6:**
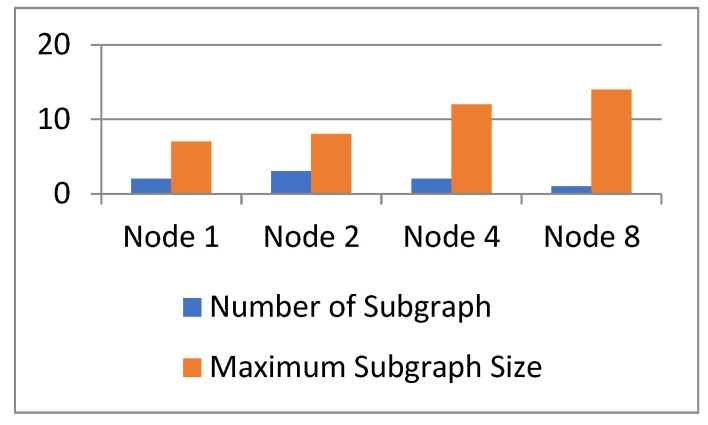
Changes in connectivity.

We delete four nodes in the network separately, and the change in network connectivity is shown in Figure 6. We can see that when node 2 is deleted, three subgraphs are generated, therefore node 2 is the most important in the network. When nodes 1 and 4 are deleted, two subgraphs are generated. However, when node 1 is deleted, the size of the largest subgraph is smaller, therefore node 1 is more important than node 4. This conclusion is consistent with the method in this paper. These results show that the method in this paper has good performance in node importance evaluation.

Spatial information network is characterized by high dynamic, its topology changes regularly in the cycle, and the structure changes very small. The algorithm proposed in this paper and the existing four node importance evaluation indexes (Degree centrality, Natural connectivity, Jump-surface node method, Mutual information method) are used to calculate the importance of each node in Figure 4 and Figure 5. The experimental data are shown in Table 4 and Table 5.

**Figure 7:**
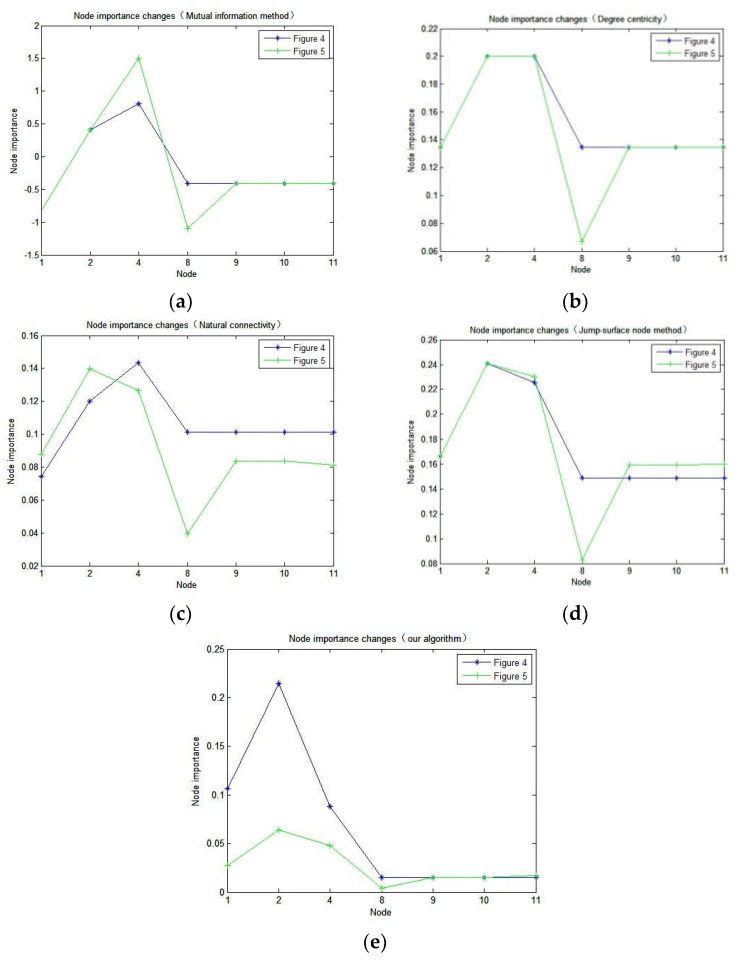
Comparison of node importance: (**a**) Mutual information method; (**b**) Degree centrality; (**c**) Natural connectivity; (**d**) Jump-surface node method; (**e**) Our method.

In order to more intuitively observe the changes of node importance before and after the network topology changes, we compared the natural connectivity, mutual information method, degree centrality, jump-surface node method, and node importance evaluation results of our algorithm, as shown in Figure 7.

#### 5.1.2. Node Importance Algorithm Discrimination Evaluation

The satellite network is characterized by high dynamics, its topological structure changes regularly during the period, and the structure changes extremely small. We use the method in this paper and the existing four node importance evaluation indicators (degree centrality, evaluation indicators based on natural connectivity, jump-surface node method, and mutual information method) to calculate the importance of nodes in Figure 4 and Figure 5. Its experimental results are shown in Figure 7.

It can be seen from Figure 7 that when the network topology in Figure 4 becomes the structure in Figure 5, the importance of node 1 calculated by degree centrality, jump-surface node method and mutual information method does not change. These methods fail to reflect that node importance changes with network topology reconfiguration. According to Figure 4, when no link is established between nodes 9 and 10, nodes 11 and 12, and nodes 13 and 14, the communication between nodes 2 and 15 needs to pass through node 1. Once node 1 fails, the spatial information network will become two unconnected symmetric subnets. When the link between node 9 and node 10, node 11 and node 12, and node 13 and node 14 is established, node 2 and node 15 can communicate through node 1. In addition, node 2 and node 15 can also communicate through the link between node 11 and node 12, therefore the importance of node 1 decreases. This conclusion is consistent with the result of the method proposed in this paper. It can be seen in Figure 7c that the importance of node 1 in Figure 5 has increased instead, indicating that the calculation result of natural connectivity is contrary to the actual situation. This is also true for node 2. At the same time, it can be found from node 11 that after the network topology changes, node 11 forms a link with node 12. When node 1 fails, node 11 can connect the left and right subgraphs. Therefore, the importance of node 11 is improved compared with that before, which is consistent with the results obtained by our method. It fully shows that the algorithm based on node importance proposed in this paper can effectively distinguish nodes with similar network topology.

### 5.2. Network Invulnerability Simulation Experiment and Analysis

As we all know, the more important a node is, the greater its influence in the network. When the more important node is attacked and fails, the communication link will also fail and the efficiency of the entire network decreases faster. We will carry out the invulnerability simulation experiment on the network topology in Figure 4 and compare our method with two existing measurable invulnerability indicators (jump-surface node method and natural connectivity).

In the previous section, we can see that node 2 is the most important in the network, node 1 is the second, node 4 is the third, and node 8 is the weakest in the network. When these four nodes fail in turn, we will simulate and calculate the invulnerability index of the spatial information network. The network invulnerability experiment results calculated by Equation (8) are shown in Table 6.

**Figure 8:**
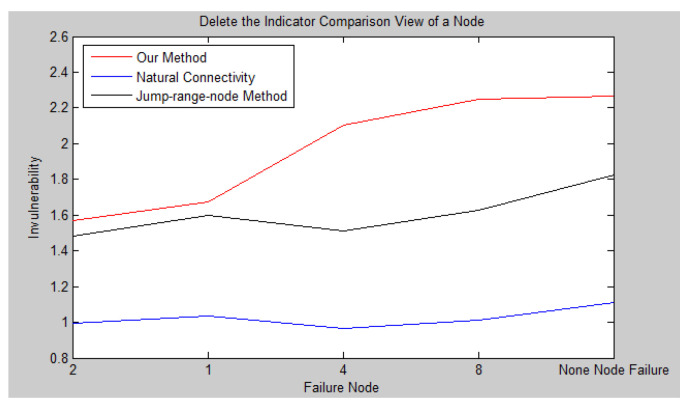
Indicator Comparison after Deleting a Node.

It can be seen from Figure 8 that when no node fails, the network’s invulnerability performance is the strongest. When any node fails, the network’s invulnerability performance decreases by varying degrees. In Figure 4, node 2 is the most important. When node 2 fails, the network’s survivability performance is the worst. It was found from the calculation results of natural connectivity that node 4 has the worst survivability when it fails, that is, the natural connectivity algorithm considers node 4 as the most important. Obviously, node 1 is more important than node 4. When node 1 fails, the survivability performance should be lower than when node 4 fails. The experimental results of the jump-surface node method are inconsistent with the actual results. In addition, node 8 has the lowest importance. When it fails, the network is the least affected and has the strongest invulnerability performance. Therefore, the entire graph should monotonically increase, which is consistent with the experimental results of this article. Based on the above analysis, we can observe that the methods in this article are more effective and accurate.

## 6. Summary

In view of the fact that non-adjacent nodes may contribute greater importance than adjacent nodes, this paper considers the influence of all adjacent nodes within the second order. First, the importance transfer probability model is proposed. At the same time, it draws on the idea of the node deletion method and uses the node importance transfer correlation matrix to determine the importance of network nodes. This method not only considers the contribution of adjacent nodes and non-adjacent nodes to the importance of the evaluated node but also considers the global importance of the node and combines the global and local ideas to make the node importance evaluation result more accurate. In addition, combined with the concept of network structure entropy, a satellite network anti-destroy performance evaluation algorithm is obtained. Simulation experiments show that the method in this paper is more accurate in evaluating the importance of nodes, and when the network topology changes, the index can effectively reflect the change. The method in this paper is also more reasonable and effective when evaluating the anti-destructive performance of the network. Therefore, the method is feasible, effective and accurate, and can be widely used in the evaluation of the importance of nodes and the invulnerability of satellite networks.

## Figures and Tables

**Table 1:** Changes in importance before and after deleting node 4.

Node	Node 3	Node 4	Node 5	Node 6	Node 7
Before deleting node 4	0.357	0.697	0.254	0.525	0.083
After removing node 4	0.238	/	0.099	0.345	0
Difference	0.118	/	0.155	0.180	0.083

**Table 2:** The effect of deleting node 1 and node 4 on node 2.

Node	Node 1	Node 2	Node 3	Node4
Original	0.140	0.128	0.357	0.697
After removing node 1	/	0.063	0.355	0.345
difference	/	0.065	0.002	0.180
After removing node 4	0.238	0.223	0.238	/
difference	0.098	0.095	0.118	/

**Table 3:** Evaluation results of node importance in Figure 2.

Degree Centricity		Natural Connectivity		Jump-Surface Node Method		Mutual Information Method		Our Method	
Node	Important Degree	Node	Important Degree	Node	Important Degree	Node	Important Degree	Node	Important Degree
2	0.2000	4	0.1436	2	0.2406	4	0.8109	2	0.2142
4	0.2000	2	0.1202	4	0.2256	2	0.4054	1	0.1064
1	0.1344	8	0.1012	1	0.1662	8	−0.4055	4	0.0884
8	0.1344	1	0.0742	8	0.1488	1	−0.8109	8	0.0152

**Table 4:** Calculation results of node importance in Figure 2.

Figure 2	1	2	4	8	9	10	11
Our method	0.1064	0.2142	0.0884	0.0152	0.0152	0.0152	0.0152
Degree centricity	0.1344	0.2000	0.2000	0.1344	0.1344	0.1344	0.1344
Natural connectivity	0.0742	0.1202	0.1436	0.1012	0.1012	0.1012	0.1012
Jump-surface node method	0.1662	0.2406	0.2256	0.1488	0.1488	0.1488	0.1488
Mutual information Method	−0.8109	0.4054	0.8109	−0.4055	−0.4055	−0.4055	−0.4055

**Table 5:** Calculation results of node importance in Figure 3.

Figure 3	1	2	4	8	9	10	11
Our method	0.0277	0.0642	0.0479	0.0037	0.0152	0.0149	0.0169
Degree centricity	0.1344	0.2000	0.2000	0.0667	0.1344	0.1344	0.1344
Natural connectivity	0.0879	0.1396	0.1266	0.0396	0.0835	0.0838	0.0810
Jump-surface node method	0.1662	0.2410	0.2304	0.0828	0.1590	0.1593	0.1597
Mutual information Method	−0.8109	0.4054	1.5040	−1.099	−0.4055	−0.4055	−0.4055

**Table 6:** Invulnerability index when nodes fail in sequence.

	Node 2	Node 1	Node 4	Node 8	No Failure Node
Our method	1.5676	1.6711	2.1025	2.2469	2.2662
Natural connectivity	0.9917	1.0376	0.9683	1.0106	1.1119
Jump-surface node method	1.4800	1.5960	1.5100	1.6280	1.8270

## Data Availability

Data is contained within the article.
